# Robust Analysis of Phylogenetic Tree Space

**DOI:** 10.1093/sysbio/syab100

**Published:** 2021-12-28

**Authors:** Martin R Smith

**Affiliations:** Department of Earth Sciences, Durham University, Durham, UK

## Abstract

Phylogenetic analyses often produce large numbers of trees. Mapping trees’ distribution in “tree space” can illuminate the behavior and performance of search strategies, reveal distinct clusters of optimal trees, and expose differences between different data sources or phylogenetic methods—but the high-dimensional spaces defined by metric distances are necessarily distorted when represented in fewer dimensions. Here, I explore the consequences of this transformation in phylogenetic search results from 128 morphological data sets, using stratigraphic congruence—a complementary aspect of tree similarity—to evaluate the utility of low-dimensional mappings. I find that phylogenetic similarities between cladograms are most accurately depicted in tree spaces derived from information-theoretic tree distances or the quartet distance. Robinson–Foulds tree spaces exhibit prominent distortions and often fail to group trees according to phylogenetic similarity, whereas the strong influence of tree shape on the Kendall–Colijn distance makes its tree space unsuitable for many purposes. Distances mapped into two or even three dimensions often display little correspondence with true distances, which can lead to profound misrepresentation of clustering structure. Without explicit testing, one cannot be confident that a tree space mapping faithfully represents the true distribution of trees, nor that visually evident structure is valid. My recommendations for tree space validation and visualization are implemented in a new graphical user interface in the “TreeDist” R package. [Multidimensional scaling; phylogenetic software; tree distance metrics; treespace projections.]

Phylogenetic analysis seeks to reconstruct historical relationships between evolving lineages, such as species, languages, or cell lines. Such analyses often identify many candidate trees, making it difficult to encapsulate the underlying phylogenetic signal. Single summary trees generated through consensus, compromise, or centroid methods (Wilkinson 1994; Nixon and Carpenter 1996) cannot communicate information about the “landscape” (Bastert et al. 2002) that trees occupy, such as the existence of tightly defined but potentially dissimilar “islands” or “terraces” (Maddison 1991).

The structure of “tree space”—formally, the metric space defined by the distances between each pair of trees in a set—can help to establish the progress of tree searches; to produce more informative summary trees; to reveal relationships within a set of optimal trees obtained from different data sets or methods; and to illuminate the posterior distribution of trees resulting from Bayesian analysis (Amenta and Klingner 2002; Stockham et al. 2002; Hillis et al. 2005; Holmes 2006; Chakerian and Holmes 2012; Whidden and Matsen 2015; Willis and Bell 2018; Wright and Lloyd 2020).

To appreciate this structure, a tree space that may have many intrinsic dimensions must be mapped into fewer: ideally two or three. However, dimensionality reduction discards information: mapping into too few dimensions will misrepresent spatial relationships. Few published studies evaluate whether a mapped tree space meaningfully depicts true tree-to-tree distances—perhaps because such distortion is deemed a theoretical rather than practical concern.

Alongside the dimensionality of a mapping, other factors known to influence the nature and utility of tree space include the method of dimensionality reduction; the means of calculating distances between trees; the specific trees used to generate the tree space; and how clusters (“islands”) of trees are identified (Hillis et al. 2005; Huang and Li 2013; Wilgenbusch et al. 2017). The methods implemented in the popular “TreeSetVis” and “treespace” software packages (Amenta and Klingner 2002; Jombart et al. 2017) are frequently used, but there otherwise seems to be little consensus as to how a method should be selected.

Here, I evaluate the behavior of eight distance metrics, four clustering approaches, and six mapping methods in the construction and interrogation of tree spaces from 128 sets of stratigraphically calibrated cladograms (Lloyd and Wright 2020). I explore the degree to which methodological decisions can materially impact the analysis and interpretation of tree space, and identify recommendations for best practice.

## Materials and Methods

Wright and Lloyd (2020) used a selection of 128 morphological data sets to demonstrate how tree space analysis can facilitate the interpretation of phylogenetic results. They estimated Bayesian trees under the Mk model of morphological evolution (Lewis 2001), partitioning data sets according to the number of observed tokens per character, and using four rate categories to describe the speed of morphological change, with each category’s mean rate drawn from the quartiles of a gamma distribution. A single MCMC run was executed in “RevBayes” (Höhna et al. 2016) for 300,000 generations. To minimize the risk of artifacts due to non-convergence of chains, I conservatively discard the first $50\%$ of Bayesian trees as burn-in, and sample 2500 of the remaining trees at uniform intervals to represent the posterior distribution.

Wright and Lloyd (2020) identified most parsimonious trees using TNT (Goloboff and Catalano 2016) under equal-weights parsimony, using exhaustive searches for data sets with $<25$ leaves, and heuristic searches for larger data sets. I include all most parsimonious trees reported, with an upper limit of 1000 trees for each data set.

I treat all trees as cladograms, discarding branch length information in order to focus exclusively on the evolutionary relationships contained within each tree. The underlying paleontological data sets contain 4–88 (median: 15) terminal taxa and 8–540 (median: 57) morphological characters, address a broad range of vertebrate and invertebrate taxa, and are each associated with stratigraphic occurrence data from the fossil record (Lloyd and Wright 2020). This broad suite of tree sets with disparate properties helps to illuminate, if incompletely, the nature of tree spaces constructed from typical morphological data sets.

Molecular data sets are not added to this sample because they cannot be directly integrated with stratigraphic information from the fossil record. Besides data type, the character of tree space may also depend on factors such as the method of inference, the signal:noise ratio, or the number of sites per taxon. While acknowledging that certain details of the results might therefore be particular to these specific data sets, this study documents the degree to which methodological decisions have the potential to influence tree space analysis.

### Distances

This study considers distances that purport to quantify the similarity of relationships between cladograms: the Robinson–Foulds (RF), matching split information (MS), phylogenetic information (PI), clustering information (CI), path (Pt), Kendall–Colijn (KC), and quartet (Q) metrics, and a new metric (SV) derived from vector representations of trees.

The RF (symmetric partition) distance (Robinson and Foulds 1981) counts the number of splits (loosely equivalent to edges or nodes) that occur in one tree but not the other, making no allowance for the existence of splits that may be almost—but not quite—identical. This distance is crude: it has a low resolution, is readily saturated, and is sensitive to the relocation of a single group within a tree (Steel and Penny 1993).

Information-theoretic distances (Smith 2020a) generalize the RF distance to account for the differing information content of differently sized splits, and to acknowledge similarities between pairs of splits that are not quite identical. These metrics construct a “matching” that pairs splits between two trees so as to maximize the amount of information that all paired splits hold in common; the amount of information not held in common gives the distance. The clustering, phylogenetic, or matching split concepts of information capture subtly different aspects of similarity between relationships.

The quartet distance (Estabrook et al. 1985) counts whether the relationships between each possible combination of four leaves are the same or different between two trees; it has a similar objective to information-theoretic distances but is slower to calculate.

Euclidian vector-based tree distances are the square root of the sum of squared differences between explicit vector representations of trees. The path distance (Steel and Penny 1993) constructs a vector such that for each pair of leaves $\{i,j\}$, the entry of the vector $e_{ij} $ is the number of edges between $i$ and $j$. For the KC (Kendall and Colijn 2016) distance with $\lambda = 0$ (which discards branch length information), $e_{ij} $ denotes the number of edges separating the common ancestor of $i$ and $j$ from the root; taxa whose most recent common ancestor is further from the root belong to a smaller taxonomic group. Setting $e_{ij} $ to the number of leaves in the smallest bipartition split containing both $i$ and $j$ provides an alternative measure of the size of a taxonomic group that is defined for unrooted trees; the Euclidian distance between such vectors defines a metric that I term the split size vector (SV) metric.

The KC metric is the only metric examined that assigns significance to the position of the root of a tree. To establish the degree to which annotating the position of the root influences the properties of tree space, all experiments with the CI distance are repeated with and without the root node labeled.

I do not consider distances that incorporate branch length information (e.g., Billera et al. 2001; Speyer and Sturmfels 2004; Garba et al. 2018), while acknowledging that these can produce “natural” tree spaces with desirable properties (Gori et al. 2016; Monod et al. 2018; Garba et al. 2021). Neither do I include “edit”-based distances, which are difficult to calculate exactly, and whose approximations exhibit undesirable properties (Smith 2020a). Other distances, which capture other aspects of tree similarity, might also be used as the basis for tree space construction: leaf-to-leaf distances (e.g., Leigh et al. 2011) emphasize branch lengths over relationships; shape metrics (e.g., Mir et al. 2013; Colijn and Plazzotta 2018) consider aspects of tree shape but not relationship information. As these distances do not denote the similarity in the evolutionary relationships implied by cladograms in any straightforward sense, I do not consider them further.

I have previously evaluated a number of tree distance metrics in their ability to assign higher distances to cladograms that denote increasingly different evolutionary relationships (Smith 2020a). In summary, these tests evaluate whether tree distances exhibit the following desirable properties: moving a single subtree a greater distance results in a greater distance to the resulting tree (“length of move”); moving a small subtree represents a smaller change than moving a larger subtree the same distance (“number of leaves moved”); few pairs of trees exhibit the maximum possible distance (“saturation”); few pairs of trees are allocated identical distance values (“sensitivity”); tree shape is not correlated with tree distance (“shape independence”); simulated clusters of trees can be recovered (“cluster recovery”); trees inferred from progressively more degraded data sets are further from the reference topology used to generate the pristine data set, whether data sets are degraded by subsampling characters (“bullseye subsampling”) or by switching character states between leaves (“bullseye miscoding”); trees separated by more subtree pruning and regrafting rearrangements tend to exhibit greater distances (“SPR rearrangement”); and random tree pairs exhibit a consistent score (“random distances interquartile range”).

The present study evaluates the KC and SV metrics against these criteria (detailed in full in Smith (2020a)), and against a new benchmark designed to explore the sensitivity of metrics to differences in tree balance. This new “balance independence” test uses 10,000 pairs of 25-leaf trees drawn from a uniform distribution. I calculate the distance between each pair of trees using each distance metric, and the degree of balance for each tree using the total cophenetic index (TCI, Mir et al. 2013), using R function $\texttt{TreeTools::TotalCopheneticIndex()}$ (Smith 2019a). Low TCI values denote a balanced tree, in which the left and right children of each node exhibit an equal number of descendants. A lack of correlation ($r^2)$ between a metric distance and the difference in TCI values indicates that a metric is independent of tree balance.

### Clustering

I identify clusters of unique tree topologies using:

- the Hartigan–Wong K-means algorithm (Hartigan and Wong 1979, R function $\texttt{kmeans()}$), with 3 random starts and up to 42 iterations;
- partitioning around medoids ($\texttt{cluster::pam()}$, Maechler et al. 2019), using 3 random starts and the algorithmic shortcuts of Schubert and Rousseeuw (2021);
- hierarchical clustering with minimax linkage (Murtagh 1983) ($\texttt{protoclust::protoclust()}$, Bien and Tibshirani 2011) (chosen after outperforming other linkage methods in initial informal analyses); and
- spectral clustering (using custom function $\texttt{TreeDist::SpectralEigens()}$ alongside $\texttt{cluster::pam()}$).

I use silhouette coefficients to calculate the optimal clustering method and number of clusters for each analysis (after Kaufman and Rousseeuw 1990). The silhouette value of a given tree compares its cohesion—its distance from each other tree within its cluster—with its separation—its distance from each tree that is not within its cluster. Values close to $+$1 denote a high proximity to other trees within its cluster; values close to $-$1 indicate proximity to trees in other clusters. The silhouette coefficient is the mean silhouette value of all trees. Following Kaufman and Rousseeuw (1990), I interpret silhouette coefficients $>$0.7 as representing “strong” structure; $>$0.5 as “reasonable” structure; $>$0.25 as “weak structure that may not be genuine”; and $<0.25$ as lacking clustering structure.

Clusterings (i.e., assignments of trees to clusters) are compared using their variation of information (VI, Meila 2007). Similar clusterings exhibit a low VI: the cluster to which a tree belongs in one clustering strongly predicts which cluster it belongs to in the other. The VI of two clusterings that each divide objects into two equally sized clusters will range from zero to two bits; the maximum possible VI decreases if clusters are uneven in size, and increases where more clusters are present in a clustering.

To evaluate whether clustering structure is preserved after mapping to two dimensions, I consider all tree sets with “reasonable” clustering structure (silhouette coefficient $>$ 0.5). I selected two mapping approaches—principal coordinates analysis (PCoA) and t-distributed stochastic neighbor embedding (t-SNE)—for detailed (and computationally expensive) investigation on the basis of preliminary analyses. After computing clusterings from distances mapped into two dimensions, I record any change to the number of clusters, and calculate the VI between clusterings computed from original and mapped distances.

### Mapping

Distances are calculated using the R (R Core Team 2021) packages “TreeDist” (Smith 2020b) and “Quartet” (Sand et al. 2014; Smith 2019b) and mapped into 1–12 dimensions using a suite of multidimensional scaling (MDS) approaches: PCoA (also termed classical MDS) (Gower 1966; R function $\texttt{stats::cmdscale()}$, R Core Team 2021); non-metric MDS with a Kruskal-1 stress function (Kruskal 1964) ($\texttt{MASS::isoMDS()}$, Venables and Ripley 2002); Sammon’s (1969) metric non-linear mapping ($\texttt{MASS::sammon()}$, Venables and Ripley 2002); curvilinear components analysis (CCA) (Demartines and Herault 1997; Sun et al. 2013) ($\texttt{ProjectionBasedClustering::CCA()}$, Thrun and Ultsch 2021), another metric MDS method; diffusion mapping (Coifman and Lafon 2006) ($\texttt{diffusionMap::diffuse()}$, Richards and Cannoodt 2019); Laplacian eigenmapping (Belkin and Niyogi 2003) ($\texttt{dimRed::embed()}$, Kraemer et al. 2018), a kernel eigenmap method; and t-SNE (van der Maaten and Hinton 2008; van der Maaten 2014) ($\texttt{Rtsne::Rtsne()}$, Krijthe 2015).

PCoA is a simple approach, which essentially rotates a high-dimensional space such that as much of the variance of the data as possible falls within the plotted dimensions (Thrun 2018). PCoA requires Euclidean distances, and converting distances between phylogenetic trees into a Euclidean space entails a loss of information (Nye 2011). To make the distances Euclidian, I follow the standard practice of adding a constant to each distance (Cailliez 1983; Jombart et al. 2017), while noting that this might distort the relative magnitude of individual distances.

Kruskal-1 and Sammon MDS mappings minimize the normalized difference between original and mapped distances, each using a separate stress function to quantify the normalized difference. In the usual case where tree distances are metrics, Sammon MDS is expected to closely resemble PCoA (Ekman and Blaalid 2011)—though it can emphasize accuracy in shorter rather longer distances (van der Maaten et al. 2009), providing a clearer depiction of local geometric features such as separation between clusters (Thrun 2018).

CCA uses a stress function that implicitly assigns points in a high number of dimensions to locations on a “manifold” that can be readily represented in fewer dimensions—akin to reconstructing original two-dimensional distances on a sheet of paper that has since been crumpled into a three-dimensional ball. This is accomplished with a stress function that penalizes distortion in distances that are short when mapped (*contra* short *original* distances, as in the Sammon stress function), allowing longer distances to deform more readily. The length scale that qualifies as “short” decreases as the mapping is refined.

Diffusion mapping is a different manifold-learning approach. Rather than minimizing a stress function, trees are represented as nodes on a graph, with each node connected to others by edges whose lengths are a function of the distances between trees. A Markov chain constructed over this graph generates a transition matrix; treating the eigenvectors of this Markov matrix as coordinates results in a low-dimensional space that, when successful, captures the main structure of the data, in particular preserving the spatial relationships of near neighbors (Coifman and Lafon 2006). Laplacian eigenmapping is a special case of diffusion mapping that emphasizes the influence of local density on the mapping, in part by connecting trees only to a number (here, 50) of their nearest neighbors in the initial graph; it is considered particularly appropriate when data contain meaningful clusters (Belkin and Niyogi 2003).

Finally, t-SNE constructs a probability distribution whereby trees that lie close to a specified tree are more probable. A low-dimensional mapping is selected in order that the equivalent treatment of mapped distances replicates this probability distribution as closely as possible.

### Distortion

To evaluate the susceptibility of a tree space to distortion on mapping, I calculate its correlation dimension (Camastra and Vinciarelli 2002), a measure of its intrinsic dimensionality—that is, the number of dimensions necessary in order to reproduce all the structure present in the tree space. I evaluate the distortion present in mappings using the product of the trustworthiness and continuity metrics (Venna and Kaski 2001; Kaski et al. 2003), calculated using R package “dreval” with $k = 10$ nearest neighbors. Trustworthiness measures the degree to which points that are nearby in a mapping are truly close neighbors; continuity, the extent to which points that are truly nearby retain their close spatial proximity when mapped. Their product gives a composite score that encapsulates both aspects of quality. I also calculate the strength of correlation (Pearson’s $r^2$ and Kendall’s $\tau )$ between original and mapped distances, which corresponds to the goodness of fit of a Shepard (1962) plot. Pearson’s $r^2$ measures the degree to which the original distance can be predicted from the magnitude of the mapped distance: it will be zero if mapped distances are random with respect to original distance, and one where the ratio between any two distances is identical before and after mapping. Kendall’s $\tau $ considers only the ranking of distances; where $\tau = 1$, tree pairs will be ranked in the same sequence whether sorted by original or mapped distances. The adequacy of PCoA mappings can be further evaluated by calculating the proportion of variation retained, or through visual examination of scree plots (Jolliffe 2002); these approaches were not systematically applied in this study.

I graphically depict stress by plotting the minimum spanning tree (MST, Gower and Ross 1969)—the shortest path connecting all trees—for 350 trees uniformly selected from the list of all Bayesian and parsimony results. Tortuous paths indicate distortion in a mapping (Anderson 1971). To quantify the distortion thus shown, I calculate the “MST extension factor,” which I define as the ratio between the mapped length of the MST and the shortest length possible for each mapping (i.e., the length of the MST calculated from mapped distances); in the absence of distortion, this ratio will be unity.

### Stratigraphic Congruence

The distribution of fossil taxa in the stratigraphic record is independent of their morphology, except insofar as both represent a single historical record of evolution (Sansom et al. 2018). The stratigraphic congruence of trees ought therefore to be reflected in the structure of any space that fully reflects the nature of the evolutionary histories implied by its constituent trees, even where the data used to assess stratigraphic fit are not used to construct the space.

Wright and Lloyd (2020) quantified stratigraphic congruence with the minimum implied gap (MIG) statistic, calculated using fossil occurrence data from the Paleobiology Database after rooting each tree on a manually specified outgroup taxon. A “gap” in the fossil record is a period of time in which a taxon is inferred to exist but is not represented by fossils. The MIG is the sum of gaps across all edges, when each node is situated at the time that minimizes gaps. A small MIG denotes a good fit with the stratigraphic record, and by implication an increased likelihood that a tree faithfully represents evolutionary history. To establish the extent to which mappings of tree space portray stratigraphic structure, I calculate the cumulative proportion of variance (adjusted $r^2)$ of stratigraphic consistency predicted by the first 1–12 dimensions of each mapping.

## Results

Six-dimensional mappings for each data set, tree distance method, and mapping method, with evaluation of clusterings and depiction of stratigraphic fit, are provided in the Supplementary Material available on Dryad at http://dx.doi.org/10.5061/dryad.kh1893240 (Smith 2021). Results obtained under the CI distance when trees were rooted do not materially differ from those when trees are treated as unrooted (Smith 2021).

### Tree Distance Metric

The results of the tests devised to compare tree distances by Smith (2020a), plus the new “balance independence” test, are presented in Table 1. Of the metrics examined, only the quartet and information-theoretic tree distances consistently reflect differences in the evolutionary relationships within trees (Table 1). Relative to these distances, Euclidian vector-based distances—the path, KC, and sv metrics—do a poor job of representing pre-defined structures in sets of trees. They are less effective at identifying known clusters of trees (Table 1, “cluster recovery”), and more often fail to assign greater distances to trees that are increasingly far from a reference tree (Table 1, “length of move,” “bullseye,” and “SPR rearrangement” tests).

**Table 1.** Performance of selected tree distance metrics against tests of tree distance behavior

Ranking	Best	2	3	4	5	Worst
Length of move: mis-orderings (0–545)	RF (0)	CID (8)	QD (94)	Path (126)	SV (188)	KC (360)
No. leaves moved: inconsistent cases (0–289)	$=$ CID (0)	$=$ Path (0)	SV (2)	KC (11)	QD (17)	RF (289)
Saturation: 11-leaf trees with max score (1–100,000)	$=$ CID (1)	$=$ QD (1)	$=$ Path (1)	$=$ SV (1)	KC (3)	RF (86,336)
Sensitivity: distinct values (100,000–1)	CID (28,939)	KC (581)	SV (541)	Path (302)	QD (200)	RF (6)
Shape independence: $r^2$ (0–1)	QD (10$^{-4})$	CID (0.0079)	RF (0.024)	SV (0.055)	KC (0.35)	Path (0.48)
Balance independence: $r^2$ (0–1)	RF (0.0001)	QD (0.0007)	CID (0.0014)	SV (0.004)	Path (0.027)	KC (0.375)
Cluster recovery: mean rank (1–25)	CID (7.2)	QD (9.8)	RF (10.9)	KC (14.1)	Path (16.3)	SV (16.9)
Bullseye subsampling successes (1000–0)	CID (650)	Path (633)	QD (626)	SV (588)	KC (573)	RF (410)
Bullseye miscoding successes (1000–0)	CID (941)	QD (809)	RF (781)	SV (709)	Path (704)	KC (598)
SPR rearrangement: Kendall’s $\tau $ (1–0)	CID (0.771)	RF (0.744)	QD (0.739)	SV (0.608)	Path (0.536)	KC (0.482)
Random distances interquartile range ($\%$ of median)	QD (1.6)	CID (1.6)	SV (2.2)	Path (18.8)	KC (35.2)	RF (n/a*)

*Note:* Parentheses denote range of possible scores for each measure (best to worst). Note that random tree pairs obtain the maximum possible RF distance, resulting in a zero interquartile range (*). Full details and results in Smith (2020a) and Smith (2021).

CID $=$ clustering information distance; KC $=$ Kendall–Colijn distance; QD $=$ quartet distance; RF $=$ Robinson–Foulds distance; SV $=$ split size vector distance.

The KC metric places a particular emphasis on differences in tree shape ($r^2 = 0.35$ for eight-leaf trees; see Table 1, “shape independence”), and thus downplays differences in the relationships between labeled leaves; $38\%$ of the variation in the KC score between pairs of 25-leaf trees can be attributed to differences in the degree of balance (Table 1, “balance independence”), compared to $<3\%$ for all other studied distances. The sensitivity of the KC metric to properties of trees that take no account of which leaf is which curtails its ability to discriminate trees based on the evolutionary relationships they imply, reducing its relevance to phylogenetic questions. The SV metric outperforms the KC metric against all but two of the examined benchmarks, but still performs poorly relative to the quartet distance and information-theoretic distances (Table 1). As such, it is difficult to see a clear case for using Euclidian vector-based distances, whose values have no straightforward interpretation, to measure the phylogenetic similarity of trees.

**Figure 1.**
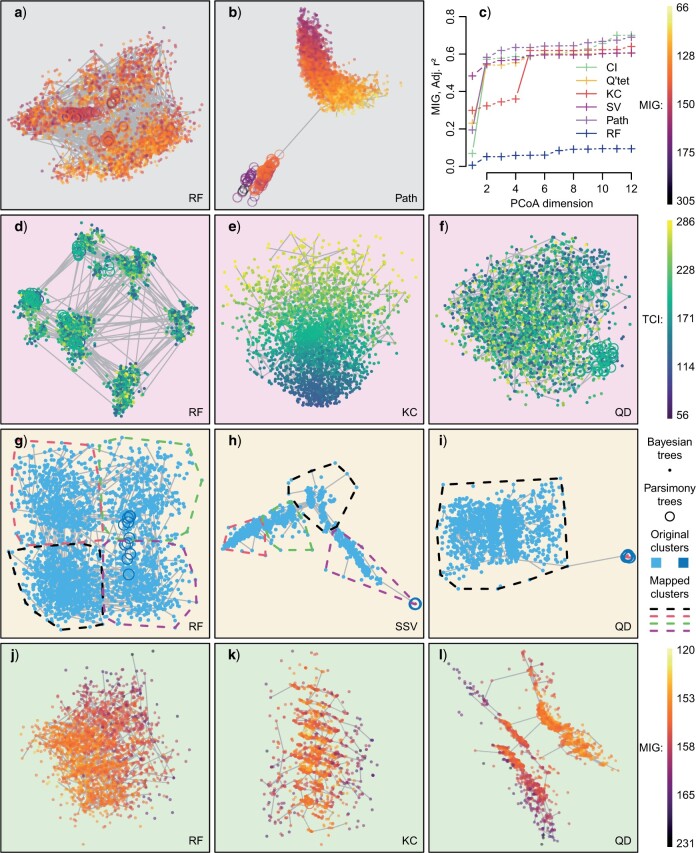
Different distances can impose tree spaces with different characteristics. First two dimensions of PCoA mappings of tree spaces, with minimum spanning tree of 350 points (solid lines). Higher dimensions depicted in Supplementary Information available on Dryad (Smith 2021). a, b) 2500 Bayesian (dots) and 100 parsimony (rings) trees from analysis of Yates (2003), colored by stratigraphic congruence (MIG, millions of years); (a) RF tree space does not exhibit clear structure; MST indicates that the two apparent clusters do not correspond to clusters in the original tree space, and that the mapping is highly distorted (MST extension factor $= 22.4$); (b) path distance tree space (MST extension factor $= 12.1$), showing stratigraphic structure and clear separation of parsimony and Bayesian trees; (c) cumulative correlation of stratigraphic fit (MIG) with first $n$ tree space axes; (d–f) 2500 Bayesian and 54 parsimony trees from analysis of Carpenter (2001); points colored by tree balance (TCI; dark $=$ balanced): (d) strong clustering in RF mapping (silhouette coefficient $= 0.70$) has no underlying basis (silhouette coefficient $= 0.040 \ll 0.2$), as suggested by tortuous minimum spanning tree (extension factor $= 33.3$); (e) vertical axis in KC mapping (MST extension factor $= 9.61$) shows clear correspondence with tree balance; (f) quartet mapping (MST extension factor $= 13.4$) faithfully represents the absence of clustering and tree balance correlation present in the original space; (g–i) trees from analysis of Fischer et al. (2016), showing (lack of) correspondence between original clusters (point color, corresponding to Bayesian vs. parsimony trees) and clusters identified from mappings (using hierarchical clustering; dashed lines $=$ convex hulls); (j–l) trees from analysis of Schoch and Milner (2008), colored by stratigraphic fit; different metrics result in spaces with different (non-clustering) structures, whose validity is supported by inspection of MST and of higher dimensions. CID $=$ clustering information distance tree space; KC $=$ Kendall–Colijn tree space; MIG $=$ Minimum implied gap; Q’tet $=$ Quartet tree space; RF $=$ Robinson–Foulds tree space; SV $=$ split size vector tree space; TCI $=$ total cophenetic index.

Because different metrics capture different aspects of tree similarity, the tree spaces they define can exhibit very different properties. The strong connection between tree shape and the KC distance means that differences in the degree of tree balance are often the primary feature of KC tree spaces, but do not characterize spaces constructed using other metrics (e.g., Fig. 1d–f). Mappings of RF spaces often stand out as particularly different to those of other spaces; in many cases, the underlying RF space lacks structures, such as clusters and correlation with stratigraphic fit, which are present in all other tree metric spaces (Fig. 1a–c, g–l; Smith 2021); though in other cases (e.g., Fig. 1d), RF mappings exhibit structure that is not evident in other spaces.

### Clusters

**Figure 2.**
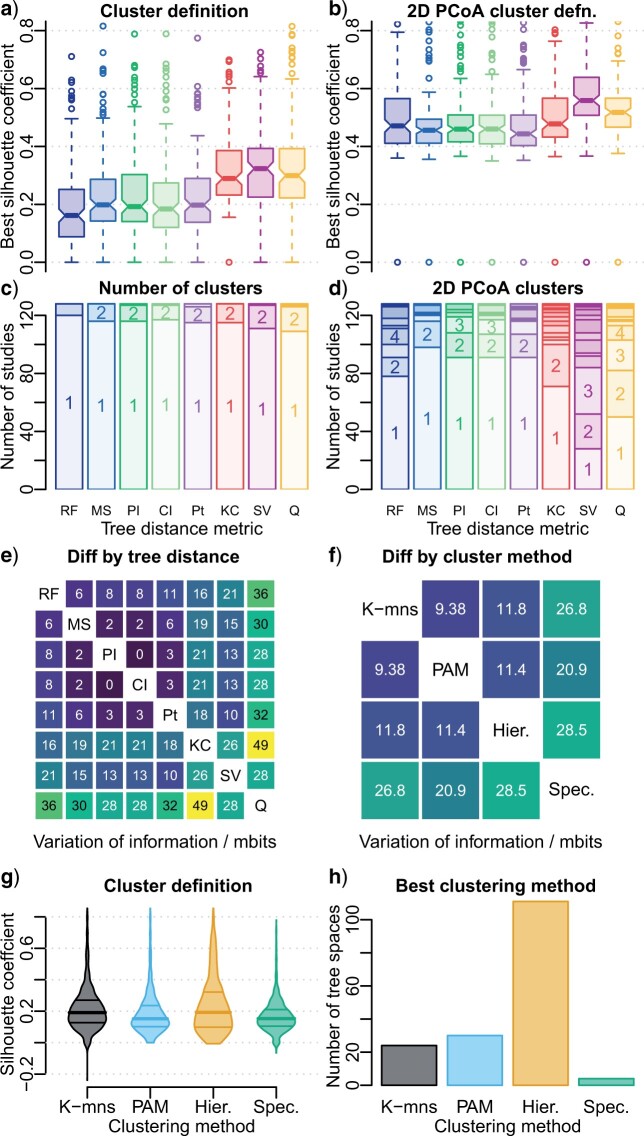
Methods for optimal clusterings. a, b) Strength of clustering (silhouette coefficient) across all 128 tree sets under each tree distance metric in (a) original tree space; (b) 2D PCoA mapping. Box plots denote median and interquartile range; strong evidence that medians differ exists where notches do not overlap. c, d) Number of clusters in optimal clustering under each tree distance method, calculated from (c) original distances; (d) 2D PCoA mapping. Tree sets lacking “reasonable” structure (i.e., silhouette coefficients $< 0.5$) are taken to exhibit a single cluster. e, f) Mean difference (variation of information) between optimal clusterings obtained under (e), each tree distance metric (f), each clustering method, from data sets exhibiting at least “reasonable” clustering structure. Brighter colors represent greater differences. g) Definition (silhouette coefficient) of optimal clustering obtained under each clustering method, summarized for all distances and tree sets. Bars denote medians and interquartile ranges. h) Method obtaining clustering with highest silhouette coefficient, across all tree spaces with at least “reasonable” clustering structure (silhouette coefficient $>$ 0.5).

If a data set displays genuine clustering structure, then it is desirable for clusters to be clearly distinguished. Tree spaces constructed on the quartet, KC, and SV metrics exhibit the most prominent clusters, whereas clustering is least defined in RF tree spaces (Fig. 2a). Better-defined clusters exhibit a higher silhouette coefficient, increasing the number of cases in which “reasonable” clustering structure (silhouette coefficient $>$ 0.5) can be identified (Fig. 2c). For tree spaces that exhibit “reasonable” clustering, the clustering solution identified is very similar (VI $ \le $ 0.01 bits) under all distance metrics except the quartet, KC, and SV metrics (VI with each other metric $ \ge $ 0.03, $ \ge $ 0.02, and $ \ge $ 0.01 bits, respectively) (Fig. 2e).

The highest silhouette coefficients are typically obtained with hierarchical clustering (Fig. 2g,h). Where “reasonable” structure is present, K-means and partitioning around medoids (PAM) tend to produce similar results to each other (VI $= 0.0094$ bits) and to hierarchical clustering (VI $= 0.011$ bits) (Fig. 2f). Spectral clustering tends to resolve clusterings that are somewhat different from those of other methods (VI $ \ge $ 0.021 bits) (Fig. 2f), often with lower silhouette coefficients; these clusters often fall below the threshold for “reasonable” structure, even in some instances where “strong” structure (silhouette coefficient $>$ 0.7) is recovered by other methods.

### Effects of Mapping

**Figure 3.**
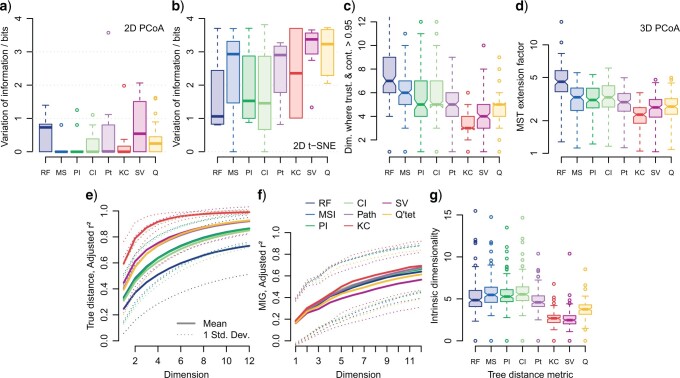
Quality of tree space mappings based on underlying tree distance method. a, b) Difference between original and mapped clusterings, in tree spaces that contain at least “reasonable” clustering structure (silhouette coefficient $>$ 0.5); (c) number of dimensions where trustworthiness and continuity are each $>$ 0.95; (d) length of minimum spanning tree relative to shortest possible in 3D PCoA mappings; increasing values indicate more distorted mappings; (e, f) cumulative correlation coefficient ($r^{2}$) between Sammon mapping axes and (e) original tree distance; (f) stratigraphic congruence (MIG); (g) correlation dimension of tree spaces. Box and whisker plots depict medians and interquartile ranges; where notches do not overlap, strong evidence exists that medians differ.

The degree of clustering is often exaggerated in two-dimensional mappings of tree space. Silhouette coefficients on clusterings calculated from mapped distances are typically higher by around 0.25 (Fig. 2a–d), with the effect that “weak” structure in the original tree space often appears “reasonable” when mapped, and “reasonable” structure often appears “strong” (for an extreme example see Fig. 1d, noting how the MST hints at a discrepancy between mapped and original distances). This said, the existence of “reasonable” structure in the original tree space does not guarantee that clustering will be evident in a two-dimensional mapping. Of the 116 tree spaces ($11\%$ of 128 data sets $\times $ eight distance metrics) with at least “reasonable” clustering structure, 19 two-dimensional PCoA mappings and 66 two-dimensional t-SNE mappings display no more than “weak” structure, meaning that genuine clusters cannot be distinguished. Even where clustering structure exists in both the original tree space and its two-dimensional mapping (97 PCoA mappings; 50 t-SNE mappings), dimensionality reduction often changes the composition of clusters markedly (VI $>$ 0.25 bits in $39\%$ of PCoA and $94\%$ of t-SNE mappings) (Fig. 1g–i). Clusterings are identical in only $53\%$ of PCoA and $6\%$ of t-SNE mappings (Fig. 3a,b). Changes in cluster composition are particularly pronounced in mappings of the Euclidian vector-based and quartet distances, and in PCoA mappings of RF distances (Fig. 3a,b).

More broadly, tree spaces defined by different metrics have different propensities for mapping. Mappings of RF tree spaces exhibit greater distortion than mappings of other spaces, reflected by lower trustworthiness and continuity metrics, higher stress, more extended MSTs, and less correlation with original distances (Fig. 3c–e). To obtain a trustworthy and continuous mapping of RF distances, it is often necessary to plot at least one dimension more than with other distance metrics (Fig. 3c). Conversely, KC tree space, and to a lesser extent the quartet, path, and SV spaces, can be mapped in a more trustworthy and continuous fashion than information-theoretic tree spaces, often attaining the same degree of distortion with one or even two fewer dimensions (Fig. 3c)—reflected by lower stress, less extension of the MST, and a higher correlation between original and mapped distances (Fig. 3c–e). Though mappings of KC, SV, and quartet tree spaces are the most faithful to the original distances, these mappings tend to exhibit a lower intrinsic dimensionality (Fig. 3g) and, for the SV and quartet spaces, a correspondingly weaker correlation with stratigraphic congruence (Fig. 3f)—suggesting that the improved mapping may reflect a simpler original tree space that fails to represent certain aspects of tree similarity.

### Mapping Method

**Figure 4.**
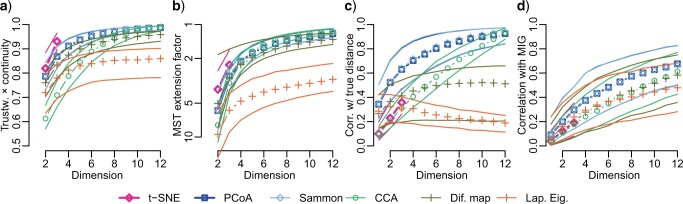
Effectiveness of mapping methods. a) Trustworthiness $\times $ continuity; (b) minimum spanning tree extension factor; (c) correlation with original distances (adjusted $r^{2}$); and (d) correlation with stratigraphic congruence (MIG, adjusted $r^{2}$). Lines depict median and interquartile range. Kruskal-1 mappings (omitted for clarity) behave equivalently to PCoA. t-SNE mapping results only available for first three dimensions.

In most cases, PCoA, Kruskal-1, and Sammon mappings of tree space differ only in small details, a recurrent theme being that Sammon maps often contain outliers plotted far from the majority of trees (Smith 2021). These methods consistently attain the highest correlation with the original distances and stratigraphic congruence, and high levels of trustworthiness and continuity (Fig. 4), indicating that these methods map the original tree space with the least distortion.

The lower correlation between other methods and original distances reflects their different motivations—for example, contraction of large distances may be seen as justified if it allows a clearer mapping of spatial relationships on a more local scale. In the case of t-SNE, this trade-off results in mappings with higher trustworthiness and continuity and with less-extended MSTs. The opposite is true for Laplacian eigenmapping, diffusion mapping, or CCA. t-SNE, Laplacian eigenmapping, and diffusion mapping each exhibit prominent and idiosyncratic structure (which may or may not correspond to structure in the original tree space), whereas a typical CCA map simply depicts a separate, approximately hyperspherical cloud corresponding to each “reasonable” cluster, with no clear evidence of any further structure (Smith 2021).

### Number of Dimensions

**Figure 5.**
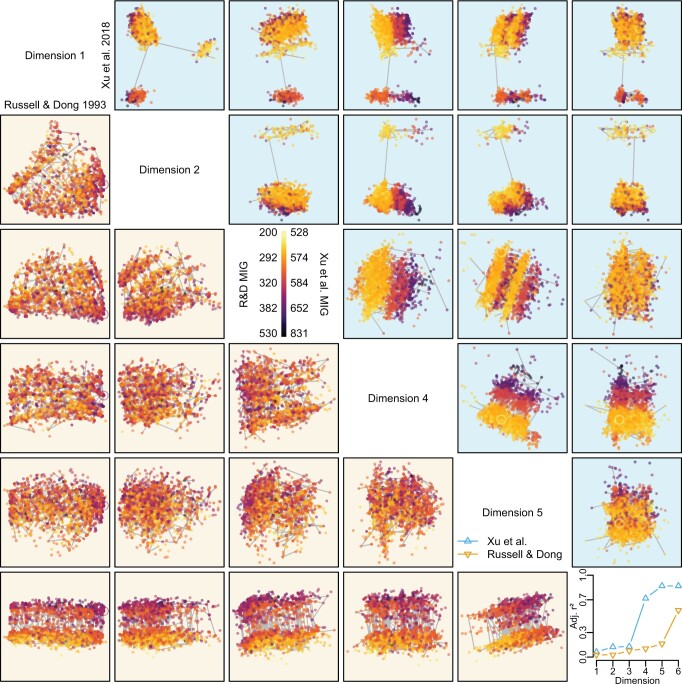
Structure “hidden” in higher dimensions. First six dimensions of phylogenetic information distance PCoA tree space, showing 2500 Bayesian trees (dots) and single most parsimonious tree (circles). Results from: bottom left, Russell and Dong (1993); top right, Xu et al. (2018). Structural features evident in higher dimensions are not apparent within the first two dimensions (top left corner), particularly with regard to stratigraphic congruence, which is strongly correlated with higher dimensions of tree space (bottom right).

RF, path, and information-theoretic tree spaces have particularly high intrinsic dimensionalities (median $ \approx $ 5; Fig. 3g). Correspondingly, in the great majority of data sets considered herein, two-dimensional mappings exhibit low ($\ll$0.95) trustworthiness and continuity values. Mapping additional dimensions depict distances more accurately and often reveals additional structure (Figs 4 and 5): it is not uncommon for a single high dimension of tree space to account for $50\%$ of the variance in stratigraphic fit (Fig. 5). In contrast, the lower dimensionalities of quartet (3.8), KC (2.7), and SV (2.5) tree spaces indicate that these spaces might often be mapped to three or even two dimensions with little distortion. But even with these metrics, two dimensions are enough to produce mappings with high values ($>$0.95) of trustworthiness and continuity only where the number of distinct tree topologies within the tree space is minimal ($<30$). A third dimension is enough to attain these values only in a minority of cases, and never in data sets containing trees with twenty or more leaves; the majority of analyses require at least four to five dimensions for a trustworthy and continuous representation (Figs 3c and 4a).

The intrinsic dimensionality of a space also reflects properties of the data sets under examination. Under all distance metrics, it is negatively correlated with the log ratio of the number of characters to the number of taxa ($r^2 = 0.1 - 0.24,P < 10^{-3}$; SupplementaryFig. S1). Dimensionality correlates positively with the total number of taxa in RF space ($r^2 \approx 0.1,P < 10^{-3})$, and negatively in quartet space ($r^2 \approx 0.1,P < 10^{-3})$, but displays no significant correlation in other metric spaces. Tree space dimensionality is positively correlated with the number of unique trees under the RF ($r^2 = 0.24,P < 10^{-3})$ and information-theoretic ($r^2 = 0.11-0.14,P < 10^{-3})$ distances, and (more weakly) the path and KC distances ($r^2 < 0.03,P = 0.03-0.04)$; but no such correlation exists under the SV and quartet distances.

## Discussion

When analyzing the distribution of phylogenetic trees, three decisions prove highly consequential: the distance metric used to construct a tree space; how clusters are identified; and how tree space is visualized.

### Distance Metric

Tree spaces are defined with reference to an underpinning distance metric. Fundamentally, a distance metric should afford smaller distances to trees that are more similar with respect to the properties under consideration—different metrics can impose profoundly different tree spaces (Fig. 1), so a tree space will only be illuminating if its underlying metric is relevant to its application.

#### Robinson–Foulds spaces

The properties of the RF distance that produce poor performance in a range of practical settings [Table 1; Steel and Penny (1993); Smith (2020a)] are particularly relevant to the construction of tree spaces: its low resolution imposes an over-quantized and thus “gappy” space; its ready saturation means that even quite similar trees can be assigned the maximum distance; and its sensitivity to the relocation of a single group or leaf means that a subset of otherwise similar trees will be allocated unrepresentatively large distances. In part, the high intrinsic dimensionality of RF tree spaces (Fig. 3g) reflects the distortions necessary to accommodate these phenomena. Correspondingly, the RF mappings analyzed in this study often contain artifacts, fail to depict structures that are apparent under other metrics, recover weaker clustering structure, and are highly distorted (Figs 1–3). As such, it is difficult to be confident that interpretations of RF tree spaces accurately represent any meaningful aspect of tree similarity.

#### Euclidian vector-based spaces

At first blush, tree spaces defined on Euclidian vector-based metrics look like promising alternatives—particularly with regard to the high fidelity of their low-dimensional mappings (Figs 1–3). The particularly low intrinsic dimensionalities of the KC and SV metrics (Fig. 3g) allow the majority of their tree space structure to be represented in three or even two dimensions (Fig. 3c, e). These two metrics also stand out for the clear definition of their clustering structure (Fig. 2a–d), even if this maps less faithfully into few dimensions (Fig. 3a, b).

However, such clustering structure often fails to correspond to artificial structure known to characterize the true distribution of trees (Table 1, “cluster recovery”). Lower intrinsic dimensionalities seem to be accomplished by downplaying phylogenetic differences between trees, resulting in simplistic spaces whose structures emphasize the contribution of tree shape. A substantial proportion of the variance in KC distances reflects the degree of tree balance (Table 1), meaning that KC tree spaces are often dominated by a single dimension that discriminates balanced from unbalanced trees (as in Fig. 1e), independently from how leaves happen to be labeled. The contribution of tree shape to the path and SV metrics, though more nuanced, results in comparable behavior. Because the relative contributions of phylogenetic and shape-based factors are not explicit in the definition of these vector-based metrics, it is difficult to disentangle their contribution to the structure of tree space. Consequently, Euclidian vector-based tree distances, and the KC metric in particular, are poorly suited to questions of evolutionary relationships.

#### Quartet and information-theoretic metrics

Though each have subtly different emphases, quartet and information-theoretic distances increase monotonically as tree topologies undergo increasing amounts of deformation (Table 1), making them inherently relevant to questions concerning the similarity of evolutionary relationships between cladograms (Smith 2020a). The MS, PI, and CI distances produce broadly similar tree spaces with similar clustering, dimensionality, and mapping characteristics (Figs. 1–3), so are treated together here. Quartet tree spaces exhibit a more pronounced clustering structure (Fig. 2a–d) and a lower intrinsic dimensionality (Fig. 3g) than information-theoretic tree spaces, meaning that they can produce more information-rich maps using fewer dimensions (Fig. 3).

In many cases, being able to obtain a tree space that discriminates clusters more readily and which requires one fewer dimension to obtain a given level of trustworthiness and continuity will more than offset the slightly poorer performance of the quartet metric against the benchmarks of Smith (2020a) and Table 1, and justify its significantly greater running time—measured in hours rather than minutes for many of the data sets examined here. On the other hand, clusters obtained using information-theoretic distances are typically rendered more faithfully in mappings. Confidence that interpreted structure genuinely characterizes the underlying trees will be greatest if its presence can be demonstrated in both quartet and information-theoretic tree spaces, which offer complementary views on the phylogenetic similarity of trees.

### Clusters

One motivation for tree space analysis is the identification of subsets of trees that are more similar with respect to the evolutionary histories they imply. This objective is most readily met when the distance from which clusters are calculated measures that property directly. Clusters identified through the visual inspection of two-dimensional tree space mappings will group trees according to *mapped* distances, which are an opaque function of original tree-to-tree distances, distorted in a manner that is particular to each mapping technique and influenced by all other tree-to-tree distances under consideration. Such clusters thus have no straightforward interpretation in their own right, except as approximations to the clustering structure imposed by the original, undistorted distances.

My results show that clusters derived from mapped distances are poor approximations to clusters based on measured distances. In the majority of RF, SV, and quartet tree spaces in which “reasonable” or better structure is present in both original and mapped spaces, clusters derived from original versus mapped distances differ substantially in their constitution (median VI $>$ 0.2 bits; Fig. 3a). Mapping a tree space into two dimensions using PCoA consistently exaggerates clustering structure, causing a mean increase in silhouette coefficient of 0.3 (Fig. 2a,b)—enough that maps may depict “reasonable” or even “strong” structure where original, undistorted distances exhibit only “weak” structure that “may not be genuine.” Correspondingly, many mappings depict multiple clusters that lack “reasonable” support in the underlying tree space (Fig. 2c,d). It is therefore inadvisable to assume that clusters interpreted from two-dimensional mappings represent genuine structure. Even if such clusters sometimes happen to group trees with certain characteristics in common, it is difficult to see how they would be preferable to a clustering derived from a direct and explicit measure of those specific characteristics.

Where a tree space does exhibit clustering structure, a secondary objective is to assign trees to clusters in a fashion that minimizes overlap between clusters, thus maximizing the silhouette coefficient. Hierarchical clustering usually performs best against this criterion (Fig. 2h), though partitioning around medoids and K-means clustering occasionally produce the best-defined clustering. Differences between the clusterings recovered by different methods tend to be relatively small (Fig. 2f), and which method is most appropriate will depend on the specific structure within a given data set and the emphases of the particular clustering methods: for example, K-means and PAM are very effective when clusters are consistent shapes or sizes, but can produce unexpected results when this assumption is violated (MacKay 2003; Hastie et al. 2009). Such factors may contribute to the poor performance of spectral clustering in these data sets (Fig. 2f–h), despite its accurate recovery of pre-defined clusters of trees in other settings (Gori et al. 2016): the geometry of these artificial tree spaces may align better with the strengths of spectral clustering. Though the use of a single clustering method is unlikely to mislead, the application of multiple methods provides additional opportunities to maximize the silhouette coefficient, and thus to better appreciate the clustering structure of a tree set.

### Visualizing Tree Spaces

Different mapping techniques have different motivations, and thus differ markedly in the structure they depict. Mapping has an order of magnitude more impact on the clustering structures perceived—the easiest aspect of structure to quantify—than the measurement of tree distance or the method of cluster detection (Figs 2e–f and 3a,b).

PCoA, Sammon, and Kruskal-1 mappings have a similar philosophy: they seek to minimize the stress induced by a mapping by minimizing a measure of distortion that penalizes mismatches between original and mapped distances. Interpretation of such mappings is straightforward: mapped distances are approximately proportional to the true distances between trees. (This does not mean that mapped *areas* are proportional to original hypervolumes—see Mammola (2019).) In line with this common principle, and despite the potential shortcomings of PCoA (Lee and Verleysen 2007), these methods often result in very similar mappings—consistent with some other results from simulated and real data sets (van der Maaten et al. 2009; Ekman and Blaalid 2011). As PCoA is significantly faster to calculate, its status as the most widely used mapping method seems justified.

CCA mapping likewise seeks to minimize stress—but the cost function employed aims not to faithfully reflect original distances, but rather to produce a “revealing representation” of the data, with an emphasis on facilitating the visual recognition of clustering (Demartines and Herault 1997). The clear depiction of clustering structure seems here to be obtained by largely discarding other aspects of tree space structure. In contrast to the results of Wilgenbusch et al. (2017), CCA-mapped distances exhibited lower correlation with original distances, and CCA mappings exhibited lower trustworthiness and continuity than PCoA, Sammon, and Kruskal-1 mappings (Fig. 4). This difference may reflect idiosyncrasies of the tree sets being examined: for example, the Wilgenbusch et al. (2017) tree sets cluster according to the gene from which trees had been inferred, so emphasizing the distinction between clusters captures relatively more of the variation in tree-to-tree distances than in data sets with weak clustering structure.

Diffusion mappings and Laplacian eigenmappings attempt to identify a lower-dimensional manifold that underlies the high-dimensional space; the poor performance of explicitly manifold-learning methods relative to other mapping techniques (Fig. 4) suggests that the sets of optimal trees examined herein are not associated with any manifold, or sample the manifold at too low a density for its inferred structure to improve mapping (Venna et al. 2010).

Finally, t-SNE obtains very high levels of trustworthiness and continuity, at the expense of a weak correlation between mapped and original distances (Fig. 4). As such, the interpretation of t-SNE mappings is not intuitive: distances between clusters, and the sizes of clusters, may not be representative; and t-SNE mappings can display apparent structure in data sets known to be homogeneous (Wattenberg et al. 2016). These properties characterize many of the t-SNE maps generated herein (see for example studies 20, 36, 89 in Smith 2021), meaning that the capacity of t-SNE mapping to represent local structure must be weighed against the danger of misinterpretation. This risk can be reduced by exploring different values of the “perplexity” and “epsilon” parameters that govern the structure of t-SNE mappings, and comparing results to a PCoA mapping.

#### Dimensionality

Tree spaces inherently exist in many dimensions. More trees tend to produce more complicated structure with more intrinsic dimensions, as previously noted by Wilgenbusch et al. (2017), and observed here under the RF and information-theoretic distances. Tree sets derived from data sets with a low character:taxon ratio also tend to exhibit higher dimensionalities, perhaps because matrices containing fewer characters constrain relationships less decisively: a broader posterior distribution of tree topologies will encompass a larger region of tree space and thus encounter more distortion when mapped (just as a map of the globe is more distorted than a cartographic map of a smaller region).

The dimensionality of tree space is also influenced by the choice of distance metric (Fig. 3). Whereas tree spaces with more intrinsic dimensions have the capacity to contain more sophisticated and instructive structure, they are harder to faithfully depict in few dimensions. This trade-off does not have a natural optimum, as the utility of a tree space is not a simple function of its dimensionality. KC and SV tree spaces obtain a low dimensionality by downplaying phylogenetic aspects of tree similarity. Although mappings produced after discarding relevant features of tree space may be less distorted, this is unlikely to compensate for the concomitant loss of information: at the extreme, a dimensionality of zero can be obtained by a metric that assigns all pairs of trees a distance of zero. On the other hand, the mere fact that more dimensions are present need not make a tree space more instructive: a metric that assigns all pairs of trees a unit distance can produce a meaningless tree space with many dimensions. Analogously, the low sensitivity and rapid saturation of the RF distance (Table 1) mean that it allocates many tree pairs identical scores, potentially increasing the number of dimensions necessary to map RF spaces without distortion (Fig. 3c–e) without a corresponding gain in utility. In contrast, the lower dimensionality of quartet tree space relative to tree spaces defined by information-theoretic distances seems not to reflect a substantial difference in how well the metrics measure the phylogenetic similarity of cladograms (Table 1). Consequently, the lower amount of distortion introduced when quartet spaces are mapped (Fig. 3c–f) is a reason to prefer this distance for visualization, so long as examination of higher dimensions of both quartet and information-theoretic spaces confirms that a low-dimensional mapping adequately summarizes structure.

Even under the quartet distance, however, the majority of data sets require more than 3 (median: 5) dimensions to attain levels of trustworthiness and continuity greater than 0.95 (Fig. 3c). Humans are less able to perceive metric distances in three-dimensional visualizations than in two dimensions (Kjellin et al. 2010), and three-dimensional displays are ineffective for estimating relative positions (Tory et al. 2006). Mappings that require multiple dimensions may thus fail in their objective of making distances easier to visualize. Wilgenbusch et al. (2017) take the more optimistic position that two-dimensional mappings capture the most important aspects of tree space structure, including clustering. In practice, I suspect that individual data sets each occupy their own position between these extremes. Although two-dimensional maps tend to exaggerate the degree of clustering—leading to the misidentification of clusters (Figs 1d, 2a–d, and 3a,b), and in some cases, the failure to depict aspects of tree space structure that are relevant to interpretation (Fig. 5)—whatever structure *is* portrayed by a two-dimensional plot can at least by deciphered at a glance, in contrast to more cognitively taxing portrayals of higher-dimensional space, which must still ultimately be perceived through the two dimensions of the retina. The potential for misinterpretation can be reduced by plotting the MST (e.g., Fig. 1d), by marking clusters that are statistically supported by the original distances, by evaluating how well a low-dimensional mapping conveys tree-to-tree distances, and by carefully examining higher dimensions for evidence of additional structure.

### Recommendations

In summary, commonly used practices are generally inadequate for the interpretation of the phylogenetic tree spaces explored herein. The KC and RF metrics do not directly measure trees’ phylogenetic similarity; their associated tree spaces are poorly suited to phylogenetic questions. Clusters identified by visual inspection of mappings are likely to misrepresent the true structure of a tree set. Two dimensions are seldom sufficient to convey the full structure of tree space, and two-dimensional mappings should be viewed with suspicion unless shown to exhibit high values of continuity and trustworthiness; a low correlation between original and mapped distances indicates that the interpretation of a mapping may require additional care.

The 128 tree sets studied herein include multiple examples where standard practice would lead to invalid conclusions. For instance, Wright and Lloyd (2020) correctly interpret the RF space of trees from Yates (2003) (Fig. 1a; cf. fig. 1C in Wright and Lloyd (2020)) as exhibiting no relationship with stratigraphic congruence—yet a strong relationship is present in all other metric spaces (see Fig. 1b and Smith (2021)). Similarity, two-dimensional mappings of Russell and Dong (1993) or Xu et al. (2018) tree spaces (Fig. 5) contain no hint of the significant correlation with stratigraphic congruence that exists in higher dimensions. Strong clustering in the mapped RF space of trees from Fischer et al. (2016) is entirely an artifact of mapping: no corresponding structure exists in the original distance matrix. These are not isolated instances, but examples that illustrate recurrent patterns evident across all examined studies; and there is no obvious reason that the tree sets analyzed here should be particularly intractable to tree space analysis. With the caveat that “landscapes” of trees selected using different optimality criteria or from different sources of data may exhibit different properties, these results raise serious concerns over the validity of previous presentations of tree spaces.

To minimize artifacts when analyzing the distribution of cladograms, I recommend that tree space analysis employs the CI or quartet distances—ideally, both. These distances are sensitive to differences in the evolutionary relationships implied by cladograms, but not to factors such as tree shape that are irrelevant to most phylogenetic questions. Information-theoretic distances (particularly the CI distance) measure the similarity between cladograms more effectively than the quartet distance and have a higher intrinsic dimensionality. Insofar as this higher dimensionality denotes a more information-rich tree space, the CI distance is well suited to the identification of clusters of trees; moreover, these clusters tend to retain their identity when mapped. On the other hand, the lower dimensionality of quartet tree space means that it suffers less distortion when mapped. Structure evident in both metric spaces might warrant additional confidence.

Except where clustering is conducted for a separate purpose (Gori et al. 2016)—for instance, when using clusterings to generate summary trees (Stockham et al. 2002)—clusters should be identified objectively from original tree distances. The clustering with the highest silhouette coefficient can be considered the best representation of the underlying structure, provided that this coefficient is high enough to indicate that the structure is meaningful ($>$0.5). Hierarchical clustering often finds the best clustering, but as the optimal method depends on the nature of clustering structure, I encourage the use of multiple clustering methods. Depicting the best clustering on mappings (as in Fig. 1g–i) reduces the potential for misinterpretation where mappings do not reflect the structure of the original tree space.

The optimal mapping method will depend on the purpose of the visualization. PCoA maps—which tend to closely resemble Sammon and Kruskal mappings, but are much faster to compute—tend to reproduce original tree-to-tree distances most faithfully, making them easy to interpret, while also depicting structure consistently (high trustworthiness and continuity); as such, they are an obvious choice for instructive mappings. Alternatively, t-SNE maps emphasize local structural relationships, though their interpretation can be counter-intuitive; whereas CCA maps depict cluster membership while downplaying other structural features.

Whichever mapping method is employed, it is important to evaluate the quality of the mapping: high ($>$0.95) values of the trustworthiness and continuity measures are desirable, as is a good correlation with original distance metrics. Even if MSTs can help to visually assess the degree of distortion, it is not possible to be confident that any apparent structure is genuine unless the quality of a mapping is explicitly documented. Of course, these metrics will be invalid, and distances misrepresented, unless plotting software is configured to plot $x$ and $y$ axes to the same scale.

These recommendations are drawn from a limited sample of morphological data sets; it is likely that tree sets obtained from different data sets using different methods will occupy tree spaces with different properties. Nevertheless, the degree to which methodological decisions can influence the interpretation of tree space represents a strong argument for conducting and documenting basic checks to establish that presented results truly represent the underlying structure of the high-dimensional tree space.

To facilitate best practice in the construction, evaluation, and interpretation of tree space, I have produced a “point-and-click” graphical interface within R—installed using $\texttt{install.packages}$$\texttt{(’TreeDist’)}$ and launched by executing the command $\texttt{TreeDist::MapTrees()}$. This software allows users to upload trees, select tree distance, mapping and clustering methods, and generate high-dimensional mappings, with real-time evaluations of mapping and clustering quality to ensure that interpretations truly reflect the underlying distribution of phylogenetic trees.

## Supplementary Material

Data available from the Dryad Digital Repository: http://dx.doi.org/10.5061/dryad.kh1893240.
